# Functional and Spectroscopic Characterization of *Chlamydomonas reinhardtii* Truncated Hemoglobins

**DOI:** 10.1371/journal.pone.0125005

**Published:** 2015-05-20

**Authors:** Chiara Ciaccio, Francisco Ocaña-Calahorro, Enrica Droghetti, Grazia R. Tundo, Emanuel Sanz-Luque, Fabio Polticelli, Paolo Visca, Giulietta Smulevich, Paolo Ascenzi, Massimo Coletta

**Affiliations:** 1 Department of Clinical Sciences and Translational Medicine, University of Roma Tor Vergata, Roma, Italy; 2 Interuniversity Consortium for the Research on the Chemistry of Metals in Biological Systems, Bari, Italy; 3 Department of Biochemistry and Molecular Biology, Faculty of Sciences, University of Córdoba, Cordoba, Spain; 4 Department of Chemistry “Ugo Schiff”, University of Firenze, Sesto Fiorentino (FI), Italy; 5 Department of Sciences, Roma Tre University, Roma, Italy; 6 National Institute of Nuclear Physics, Roma Tre University Section, Roma, Italy; 7 Interdepartmental Laboratory of Electron Microscopy, Roma Tre University, Roma, Italy; University of Hyderabad, INDIA

## Abstract

The single-cell green alga *Chlamydomonas reinhardtii* harbors twelve truncated hemoglobins (Cr-TrHbs). Cr-TrHb1-1 and Cr-TrHb1-8 have been postulated to be parts of the nitrogen assimilation pathway, and of a NO-dependent signaling pathway, respectively. Here, spectroscopic and reactivity properties of Cr-TrHb1-1, Cr-TrHb1-2, and Cr-TrHb1-4, all belonging to clsss 1 (previously known as group N or group I), are reported. The ferric form of Cr-TrHb1-1, Cr-TrHb1-2, and Cr-TrHb1-4 displays a stable 6cLS heme-Fe atom, whereas the hexa-coordination of the ferrous derivative appears less strongly stabilized. Accordingly, kinetics of azide binding to ferric Cr-TrHb1-1, Cr-TrHb1-2, and Cr-TrHb1-4 are independent of the ligand concentration. Conversely, kinetics of CO or NO_2_^−^ binding to ferrous Cr-TrHb1-1, Cr-TrHb1-2, and Cr-TrHb1-4 are ligand-dependent at low CO or NO_2_^−^ concentrations, tending to level off at high ligand concentrations, suggesting the presence of a rate-limiting step. In agreement with the different heme-Fe environments, the pH-dependent kinetics for CO and NO_2_−binding to ferrous Cr-TrHb1-1, Cr-TrHb1-2, and Cr-TrHb1-4 are characterized by different ligand-linked protonation events. This raises the question of whether the simultaneous presence in *C*. *reinhardtii* of multiple TrHb1s may be related to different regulatory roles.

## Introduction

Based on phylogeny, the globin superfamily contains three lineages: (*i*) flavohemoglobins and single domain globins, (*ii*) protoglobins and globin coupled sensors, and (*iii*) truncated hemoglobins (TrHb). Members of the globin superfamily belong to two structural classes, namely (*a*) the classical 3-on-3 α-helical sandwich (*i*.*e*., flavohemoglobins, single domain globins, protoglobins, and globin coupled sensors), and (*b*) the 2-on-2 α-helical sandwich, which is a sub-editing of the classical 3-on-3 α-helical fold (*i*.*e*., TrHbs). The function of globins in unicellular organisms is primarily (pseudo-)enzymatic, with O_2_ transport and storage being specialized functions associated with the evolution of metazoans [1–8].

TrHbs are widely distributed in bacteria and plants, and have been also found in some unicellular eukaryotes. TrHbs branch into three classes, designated 1 (or group N or group I), 2 (or group O or group II), and 3 (or group P or group III). TrHbs belonging to classes 1 and 2 are separated into two and four subgroups, respectively, whereas TrHbs belonging to class 3 are grouped in a single subclass displaying a high level of overall conservation [7,9]. The members of the three classes adopt a simplified globin fold based on four main α-helices. Unlike other globins, the heme cavity of TrHbs shows a great variability between groups and subclasses, particularly on the distal side. Except for the proximal His(F8) residue bound to the heme iron and the Phe(B9)-Tyr(B10) pair, commonly found in the distal cavity, the other residues constituting the heme surrounding are not conserved in TrHbs [3]. Such a variability in the protein matrix reflects different ligand binding properties of members from different groups of TrHbs, thus suggesting biological functions related to O_2_/NO chemistry [7].

Some bacteria, plants, and unicellular eukaryotes produce multiple TrHbs belonging to different classes. From the evolutionary viewpoint, it has been postulated that the members of classes 1 and 3 evolved from the class 2 gene as the result of duplications and transfer events [1–8]. Remarkably, the occurrence of several TrHbs in a single organism suggests a pivotal role in diverse physiological functions, including protection against oxidative damage by ferrous nitrosylated TrHb, NO detoxification by ferrous oxygenated TrHb, NO formation from NO_2_^−^ by deoxygenated ferrous TrHb, and peroxynitrite scavenging by ferric TrHb; each process then depends on the redox and ligation state of the heme-protein [10–13].

*Chlamydomonas reinhardtii* is a unicellular green alga which has provided a valuable reference system for the investigation of fundamental biological functions associated with both the plant and the animal lineages. This is not only due to the ease of handling and manipulating this organism in the laboratory, but also to the peculiar evolutionary history of the genus *Chlamydomonas*. It has retained features, like the ciliary and flagellar functions, that are present in animals but have been lost in higher plants, combined with a remarkable conservation of the photosynthetic apparatus [14]. Interest on *C*. *reinhardtii* was considerably boosted by the publication of its complete genome sequence in 2007, which allowed the identification of novel gene functions and pathways [15]. In *C*. *reinhardtii*, twelve TrHbs have been recently reported [16]. Cr-TrHb1-1 has been linked to the nitrogen assimilation pathway since it binds NO in both the ferric or ferrous state and displays an efficient NO dioxygenase activity [17]; on the other hand, Cr-TrHb1-8 has been postulated to be part of a NO-dependent signaling pathway [16].

In the present study, a detailed characterization of the spectroscopic and reactivity properties of three TrHbs from *C*. *reinhardtii* (*i*.*e*., Cr-TrHb1-1, Cr-TrHb1-2, and Cr-TrHb1-4), all belonging to class 1 (previously known as group N or group I), is reported. The rationale behind this choice is that the expression of these truncated Hbs has been shown to be modulated by the nitrogen source and to depend on NIT2, a transcription factor required for nitrate assimilation genes expression [18]. Remarkably, the ferric form of Cr-TrHb1-1, Cr-TrHb1-2, and Cr-TrHb1-4 displays a stable poorly reactive six-coordinated low-spin (6cLS) heme, whereas the ferrous forms are more reactive toward exogenous ligands, suggesting a weaker or absent hexa-coordination of the heme-Fe atom. Accordingly, ferrous Cr-TrHb1-1, Cr-TrHb1-2, and Cr-TrHb1-4 catalyze the NO_2_^−^ conversion to NO very efficiently, representing a viable pathway for NO generation under anaerobic conditions.

## Materials and Methods

### Recombinant protein expression and purification

THB (Truncated Hemoglobin) cDNAs were amplified using differents primers and cloned in pSpark cloning vector (DH5αF’ E. coli strain); in particular, cDNAs for Cr-TrHb1-1 (THB1), for Cr-TrHb1-2 (THB2), and Cr-TrHb1-4 (THB4) were amplified by PCR with primers THB1-KpnIfw 5’-GGTACCATGGCCGCCGATACCGC-3’ and THB1-HindIIIrev 5’-AAGCTTCTAATTGGCGGCGCCGGCCT-3’, THB2-KpnIfw 5’-GGTACCATGTCGCCCGCGCCTGAAACTGT-3’ and THB2-HindIIIrev 5’-AAGCTTTTACACCCGCTCAAAGATGACG-3’ and THB4-KpnIfw 5’-GGTACCATGGCAGCAGTTAATAACGGT-3’ and THB4-HindIIIrev 5’-AAGCTTTTACATGATTGTGCAGCACGCTGG-3’.

After this first phase, pQE80L (Qiagen, Hilden, Germany) was used as expression vector. This plasmid was used to transform the *E*. *coli* strain C41 (6 Histidine residues in the *N*-terminal for the protein expression). THBs induction was performed overnight at 37°C with 100 μM IPTG, and purification was carried out with a Ni-nitrilotriacetic acid (Ni-NTA) matrix, as recommended by the supplier (Qiagen, Hilden, Germany), under native conditions at 4°C. Cells were grown aerobically in LB medium. After centrifuging (10,000 × *g* for 5 min at 4°C), cells were re-suspended in the extraction buffer (Tris-HCl 50 mM, pH 8; NaCl 300 mM; 5% glycerol; imidazole 10 mM pH 7). The cells lysed by ultrasonication were centrifuged at 15,000 × *g* for 10 min at 4°C in order to remove remaining cells. The supernatant is the crude extract from which the purification of the protein was carried out.

### Sample preparation

All chemicals were of analytical or reagent grade and were used without further purification. All measurements were performed at room temperature in a 0.1 M phosphate buffer solution between pH 6.8 and 7.0. For kinetic experiments, the ferrous form of Cr-TrHb1-1, Cr-TrHb1-2, and Cr-TrHb1-4 was obtained by adding a freshly prepared sodium dithionite (Fluka Chemicals, Buchs, Switzerland) solution (to a final concentration of 3 mM) to the ferric deoxygenated protein solutions. The CO complexes of Cr-TrHb1-1, Cr-TrHb1-2, and Cr-TrHb1-4 for the resonance Raman (RR) experiments were prepared by equilibrating the protein solutions with 1 atm ^12^CO (Rivoira, Milan, Italy) or ^13^CO (FluoroChem, Hadfield, UK) and finally adding sodium dithionite (to a final concentration of 3 mM).

### Spectroscopic characterization

Electronic absorption spectra of Cr-TrHb1-1, Cr-TrHb1-2, and Cr-TrHb1-4 were obtained with a double-beam spectrophotometer (Varian Cary 5, Palo Alto, Ca, USA). RR spectra were recorded by excitation at 413.1 nm (Kr+ laser, Coherent, Innova 300C, Santa Clara, Ca, USA) and 441.6 nm (He-Cd laser, Kimmon IK4121R-G, Tokyo, Japan) at room temperature. The backscattered light from a slowly rotating 5 mm NMR was collected and focused into a computer-controlled triple spectrometer (consisting of two Acton Research SpectraPro 2300i working in the subtractive mode, and a SpectraPro 2500i in the final stage with a 3600 grooves/mm grating. 1800 grooves/mm grating has been used for the experiment of the CO complexes in the 1800–2000 cm^-1^ region, Acton, Ma, USA), equipped with a liquid-nitrogen cooled CCD detector (Roper Scientific Princeton Instruments, Trenton, NJ, USA). Absorption spectra were measured both prior to and after RR measurements at room temperature to ensure that no degradation had taken place under the experimental conditions used. All RR measurements were repeated several times under the same conditions to ensure reproducibility. To improve the signal-to-noise ratio, a number of spectra were accumulated and summed only if no spectral differences were noted. All spectra were baseline corrected. The RR spectra were calibrated with indene, carbon tetrachloride, pyridine, *n*-pentane, acetone, and acetonitrile as standards to an accuracy of 1 cm^−1^ for intense isolated bands.

### Kinetic measurements

Kinetic measurements have been carried out by rapid-mixing experiments using the SX18.MV stopped-flow apparatus (Applied Photophysics, Salisbury, UK) equipped with a diode array for spectra acquisition over 1 ms time range. Ligand binding experiments have been undertaken mixing Cr-TrHb1-1, Cr-TrHb1-2 or Cr-TrHb1-4 (4×10^–6^ M) with varying concentrations of different reactants (*i*.*e*., sodium azide, CO or NO_2_^−^) at 20°C and following the spectral evolution over the 380–450 nm wavelength range.

The pH-dependence of the CO binding kinetics and nitrite reductase activity of ferrous Cr-TrHb1-1, Cr-TrHb1-2 or Cr-TrHb1-4 was carried out by mixing in the stopped-flow apparatus the reduced heme-protein solutions (in 1 mM phosphate buffer pH 7.0) with ligand solutions (in 0.15 M phosphate and/or acetate buffer after 1:1 mixing) at the desired pH values and following the spectral evolution over the 380–450 nm wavelength range. Progress kinetic curves at selected wavelengths were analyzed according to Eq 1:

ODobs=OD0±∑i=1i=nΔODi⋅exp(−ki⋅t) (Equation 1)

where *OD*_*obs*_ is the observed optical density at a selected wavelength and at a given time interval, *OD*_*0*_ is the optical density at *t* = 0, *n* is the number of exponentials, Δ*OD*_*i*_ is the optical density change associated to the exponential *i*, ^*i*^ *k* is the rate constant of the exponential *I*, and *t* is the time.

The oxygen pulse method has been employed to measure the kinetics of O_2_ dissociation. This approach consists in mixing unliganded ferrous Cr-TrHb1s in the presence of sodium dithionite (at a final concentration of 3 mM) with the oxygenated buffer; in this way, the heme iron is always in a fully reduced state and autooxidation rate has not to be considered. After mixing, there is an initial O_2_ binding to the heme iron, followed immediately (within few tens of milliseconds) by the O_2_ dissociation, due to the very rapid destruction of O_2_ by sodium dithionite.

### Data analysis

Kinetic data were analyzed using the MatLab program (The Math Works Inc., Natick, MA, USA). The results are given as mean values of at least four experiments plus or minus the corresponding standard deviation.

### Sequence analysis and molecular modeling

The molecular models of Cr-TrHb1-1, Cr-TrHb1-2, and Cr-TrHb1-4 were built using the pipeline implemented in the *ab initio* molecular modeling package I-TASSER [19]. I-TASSER identifies template proteins which are predicted to display a fold similar to the protein of interest using several different threading approaches. Subsequently, structure fragments derived from the templates are assembled by replica-exchange Monte Carlo simulations and the resulting models are refined by iteratively optimizing the free energy and the global topology of the models [20]. The best template identified by I-TASSER for Cr-TrHb1-1 and Cr-TrHb1-4 was the structure of the *Chlamydomonas eugametos* TrHb1 (PDB code: 1DLY) [9], while for Cr-TrHb1-2 the best template was the structure of the *Tetrahymena piriformis* TrHb1 (PDB code: 3AQ5) [21].

## Results and Discussion

### Cr-TrHb1s molecular models

**Fig 1:**
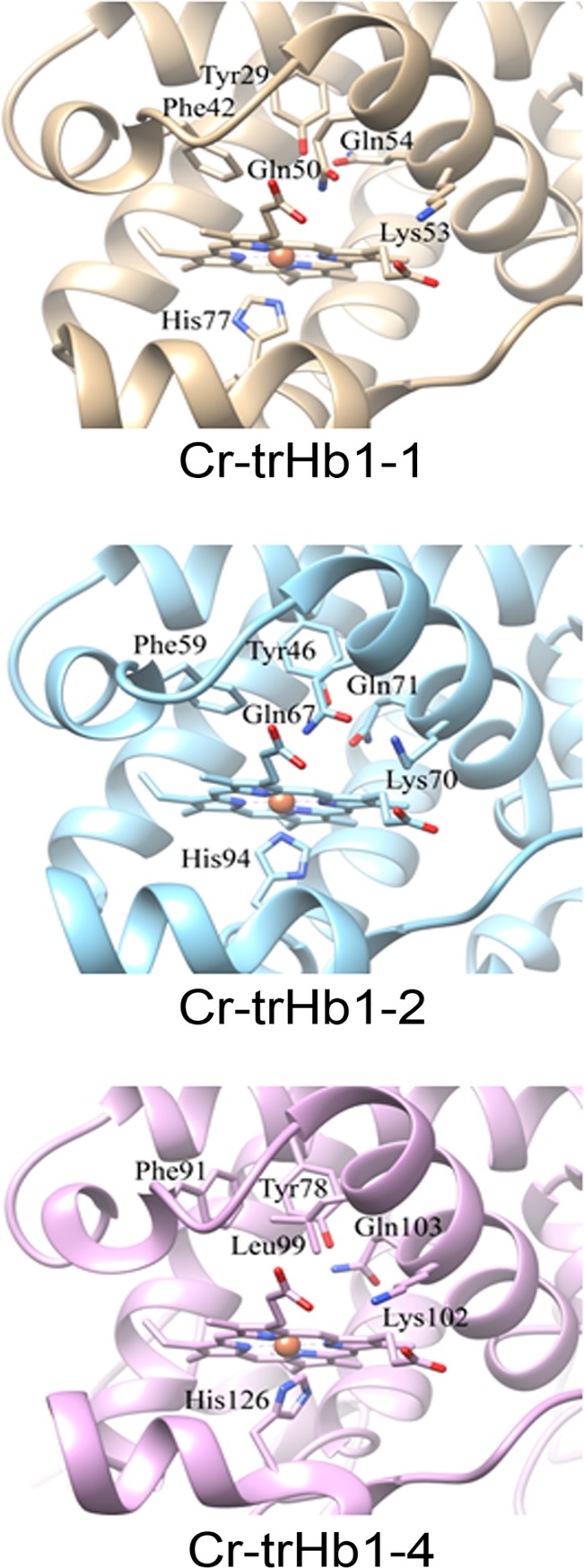
Schematic representation of the heme environment in the structural models of Cr-TrHb1-1, Cr-TrHb1-2, and Cr-TrHb1-4. For clarity, the only side chains shown are those of the proximal His ligand, of the residues lining the heme distal cavity, and of the Lys residue hypothesized to be the sixth iron ligand in the absence of exogenous ligand [17].

Analysis of the molecular models of Cr-TrHb1-1, Cr-TrHb1-2, and Cr-TrHb1-4 indicate that, in spite of the fact that in the I-TASSER threading procedure different templates have been selected for Cr-TrHb1-1, Cr-TrHb1-2, and Cr-TrHb1-4, the overall fold of the core region of the three proteins is very similar (see S1 Fig), and they all belong to group I TrHb1s [22]. Exceptions are represented by the *N*-terminal extension of Cr-TrHb1-2, predicted to form an α-helix which partially hinders the access to the heme distal cavity, and the *N*- and *C*-terminal extensions of Cr-TrHb1-4, predicted to be largely disordered. Fig 1 focuses on the heme environment of the molecular models of Cr-TrHb1-1, Cr-TrHb1-2, and Cr-TrHb1-4. In all three models, the residue closest to the heme iron on the distal side is a Tyr residue (*i*.*e*., Tyr29, Tyr46, and Tyr78 in Cr-TrHb1-1, Cr-TrHb1-2, and Cr-TrHb1-4, respectively), as also observed in the *Chlamydomonas eugametos* TrHb1 structure [9]. The other residues lining the distal heme cavity appear also to be conserved (Fig 1), with the exception of Leu99 in Cr-TrHb1-4, since in the orthologous position a Gln residue is observed in Cr-TrHb1-1 and Cr-TrHb1-2 (*i*.*e*., Gln50 and Gln67, respectively). Interestingly, also the Lys residue proposed by Johnson and coworkers [17] to be the sixth iron ligand in the absence of an exogenous ligand (*i*.*e*., Lys53, Lys70 and Lys102 in Cr-TrHb1-1, Cr-TrHb1-2, and Cr-TrHb1-4, respectively) appears to be conserved and similarly positioned in all three proteins (Fig 1). In this regard, it must be noted that in the models the distance between the Nε of this Lys residues and iron is approx. 10 Å. However, a simple reorientation of the sidechain, without any backbone conformational change, results in a distance lower than 4.0 Å (data not shown). Further, the α-helix B in which this Lys residue is located is joined to the rest of the structure, on the *C*-terminal side, by the highly flexible Gly-Gly-Ser-Gly-Ala sequence. Thus a small reorientation of the α-helix B could lead the Lys residue to the right distance to coordinate the heme-iron.

### Spectroscopic properties

#### Fe(III) form

**Fig 2:**
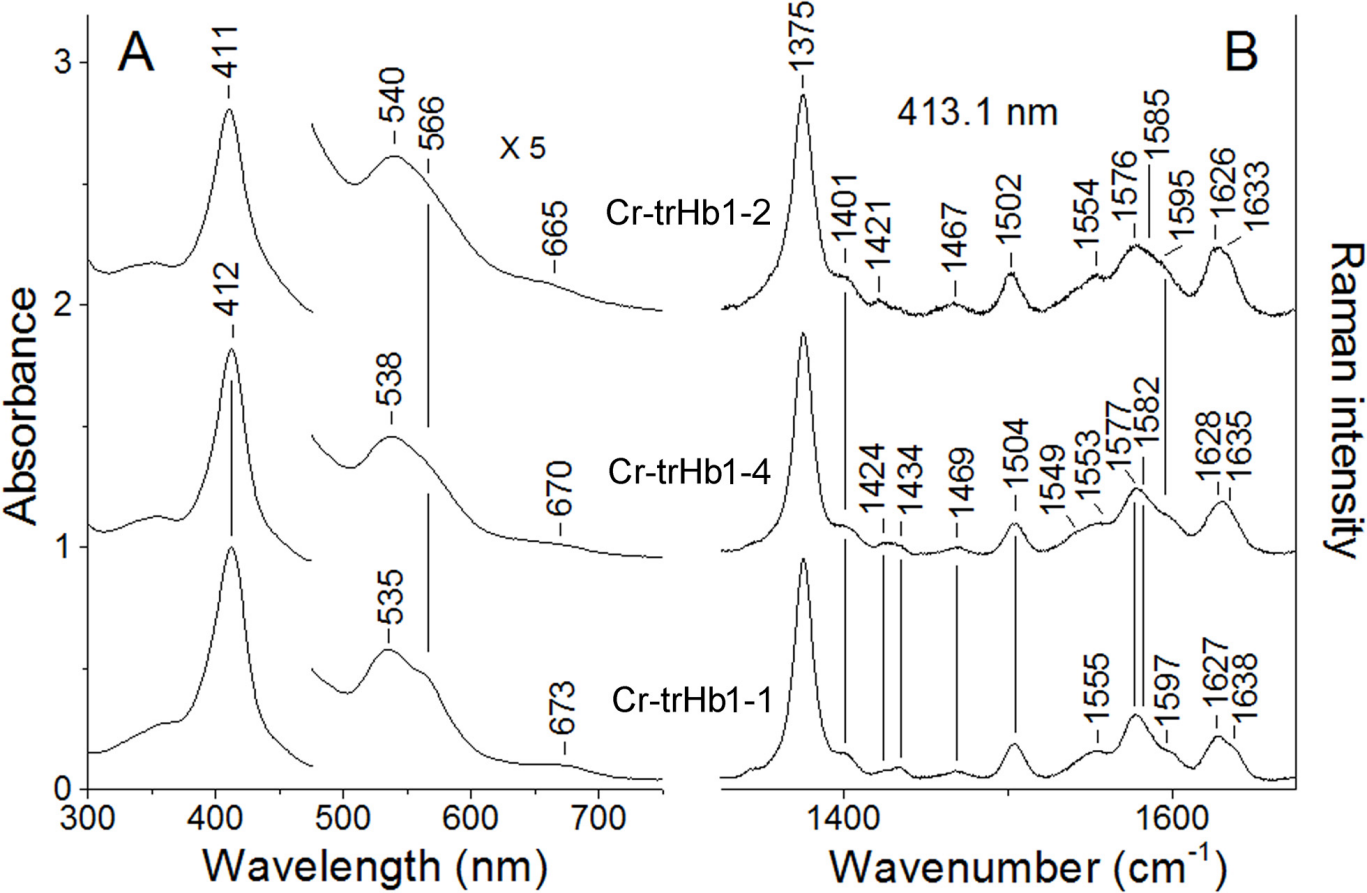
Absorption spectra (*panel A*) and resonance Raman spectra in the high-frequency region (*panel B*) of ferric Cr-TrHb1-1, Cr-TrHb1-2, and Cr-TrHb1-4. The resonance Raman spectra were obtained with excitation wavelength at 413.1 nm. Experimental conditions: 1 cm^−1^ resolution and 10 mW laser power at the sample; the total accumulation time was 10 min for Cr-TrHb1-2, 32 min for Cr-TrHb1-4, and 24 min for Cr-TrHb1-1. Spectra were obtained between pH 6.8 and 7.0 at 20°C. Spectra have been shifted along the ordinate axis to allow a better visualization.

The absorption spectra of Cr-TrHb1-1, Cr-TrHb1-2, and Cr-TrHb1-4 (Fig 2, panel A), obtained between pH 6.8 and 7.0 at 20.0°C, are characteristic of a 6cLS heme, with a Soret band at 411–412 nm, and in the visible region bands at 535 nm (for Cr-TrHb1-1), 540 nm (for Cr-TrHb1-2), and 538 nm (for Cr-TrHb1-4). The spectrum of Cr-TrHb1-4 has also been obtained at pH 7.8 (not shown), which displays a red-shift of the bands in the visible region, suggesting the formation of a 6cLS displaying an OH^−^ ligand bound to the heme-Fe atom. The bands around 665–670 nm (observed in all the UV-Vis spectra) are probably due to the presence of an impurity or heme *d* [23].

**Fig 3:**
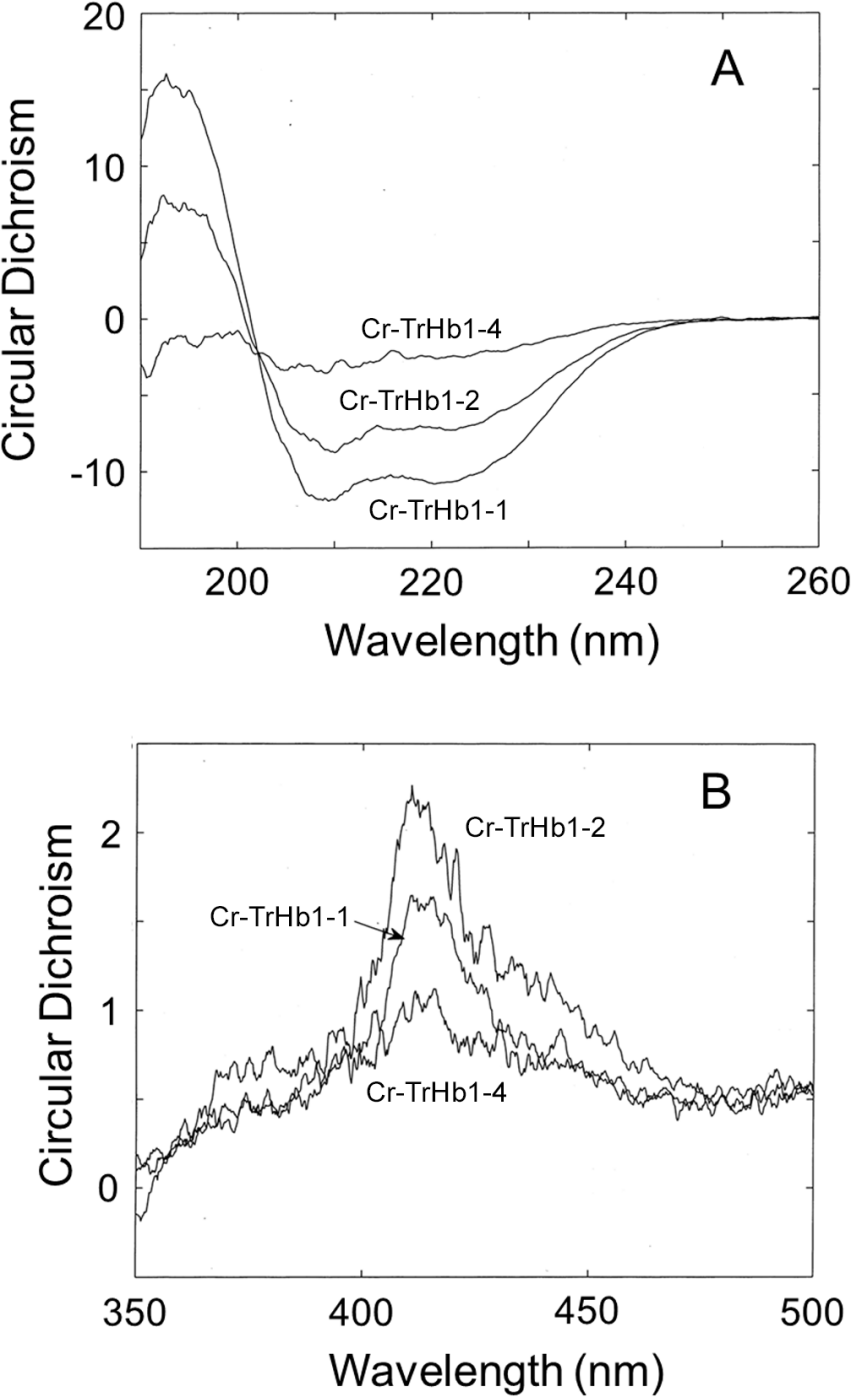
Circular dichroism spectra of ferric Cr-TrHb1-1, Cr-TrHb1-2, and Cr-TrHb1-4 in the near-UV region (*panel A*) and in the Soret region (*panel B*). Spectra were obtained between pH 6.8 and 7.0 at 20°C.

In agreement with the UV-Vis spectra, the RR spectra of Cr-TrHb1-1, Cr-TrHb1-2, and Cr-TrHb1-4 (Fig 2, panel B) are characteristic of 6cLS heme. The spectra are very fluorescent and therefore they have been background subtracted. The spectra are very similar to each other with small frequency differences; in particular, Cr-TrHb1-1 and Cr-TrHb1-4 show core size marker bands which are 2–3 cm^-1^ up-shifted, as compared to the corresponding bands of Cr-TrHb1-2, indicating a stronger endogenous sixth ligand of the heme-Fe atom. In fact, due to the correlation between the RR frequency and the size of the porphyrin core these bands are indicative of oxidation, spin and coordination states of the heme iron [23]. Fig 3 (panel A) shows CD spectra of Cr-TrHb1-1, Cr-TrHb1-2, and Cr-TrHb1-4 in the near-UV region, from which it emerges that Cr-TrHb1-1 displays the highest percentage of α-helix (as indicated by the more pronounced well at 222 nm), whereas Cr-TrHb1-4 shows a quite disordered structure, suggesting that it is less structured. This conclusion is reinforced by the spectra in the Soret region, (see Fig 3, panel B), since a well defined ellipticity peak was observed in the ferric form of Cr-TrHb1-1 and Cr-TrHb1-2, while the ellipticity of Cr-TrHb1-4 is much less pronounced, indicating that the heme-Fe atom is in a more symmetric environment (*i*.*e*., it displays a more stable 6cLS coordination) [24].

#### Fe(II) form

**Fig 4:**
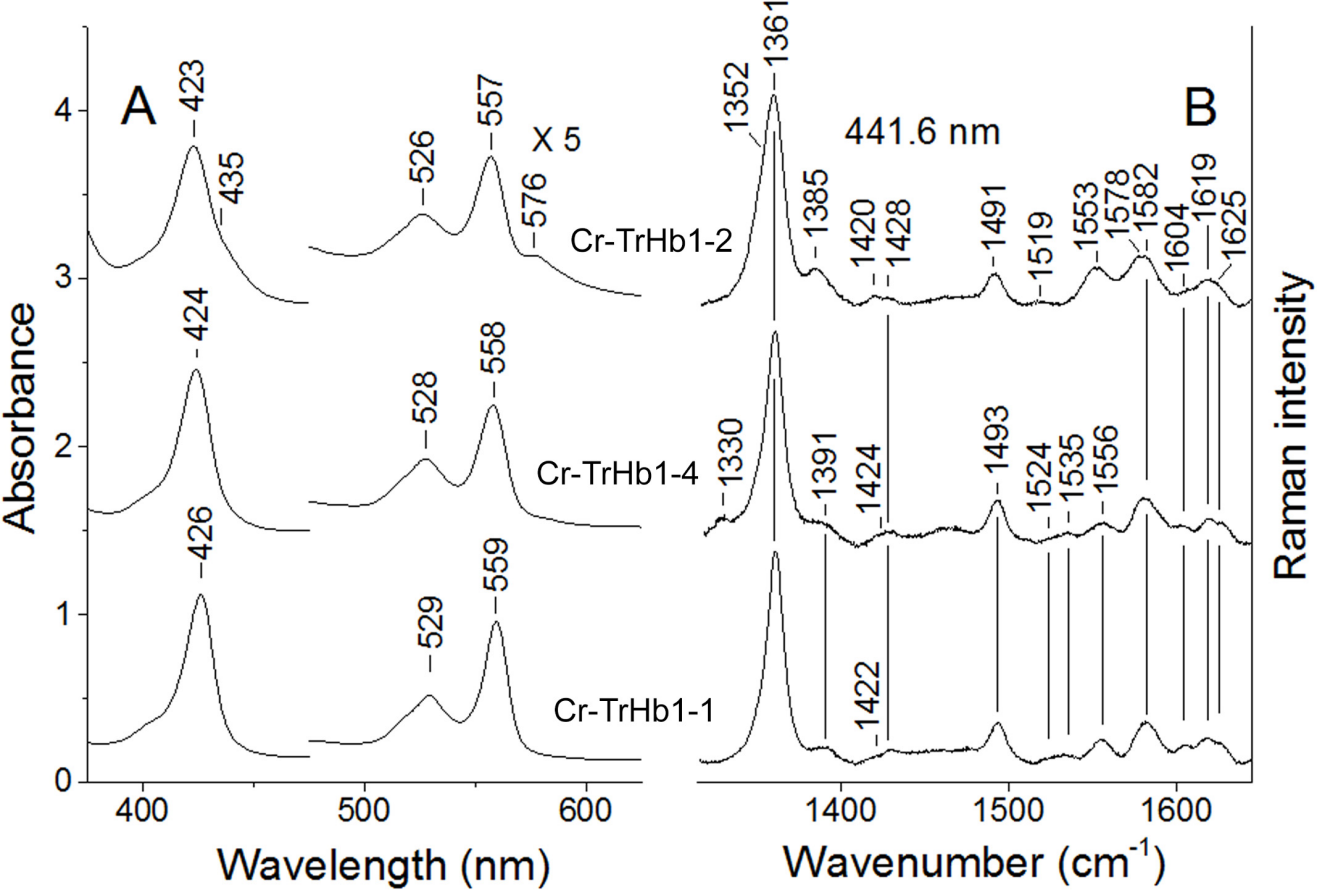
Absorption spectra (*panel A*) and resonance Raman spectra in the high-frequency region (*panel B*) of ferrous Cr-TrHb1-1, Cr-TrHb1-2, and Cr-TrHb1-4. The resonance Raman spectra were obtained with excitation wavelength at 441.6, 1. Experimental conditions: 1 cm^−1^ resolution and 15 mW laser power at the sample; the total accumulation time was 20 min for Cr-TrHb1-2, 15 min for Cr-TrHb1-4, and 10 min for Cr-TrHb1-1. Spectra were obtained at pH 7.0 and 20°C. Spectra have been shifted along the ordinate axis to allow better visualization.

**Fig 5:**
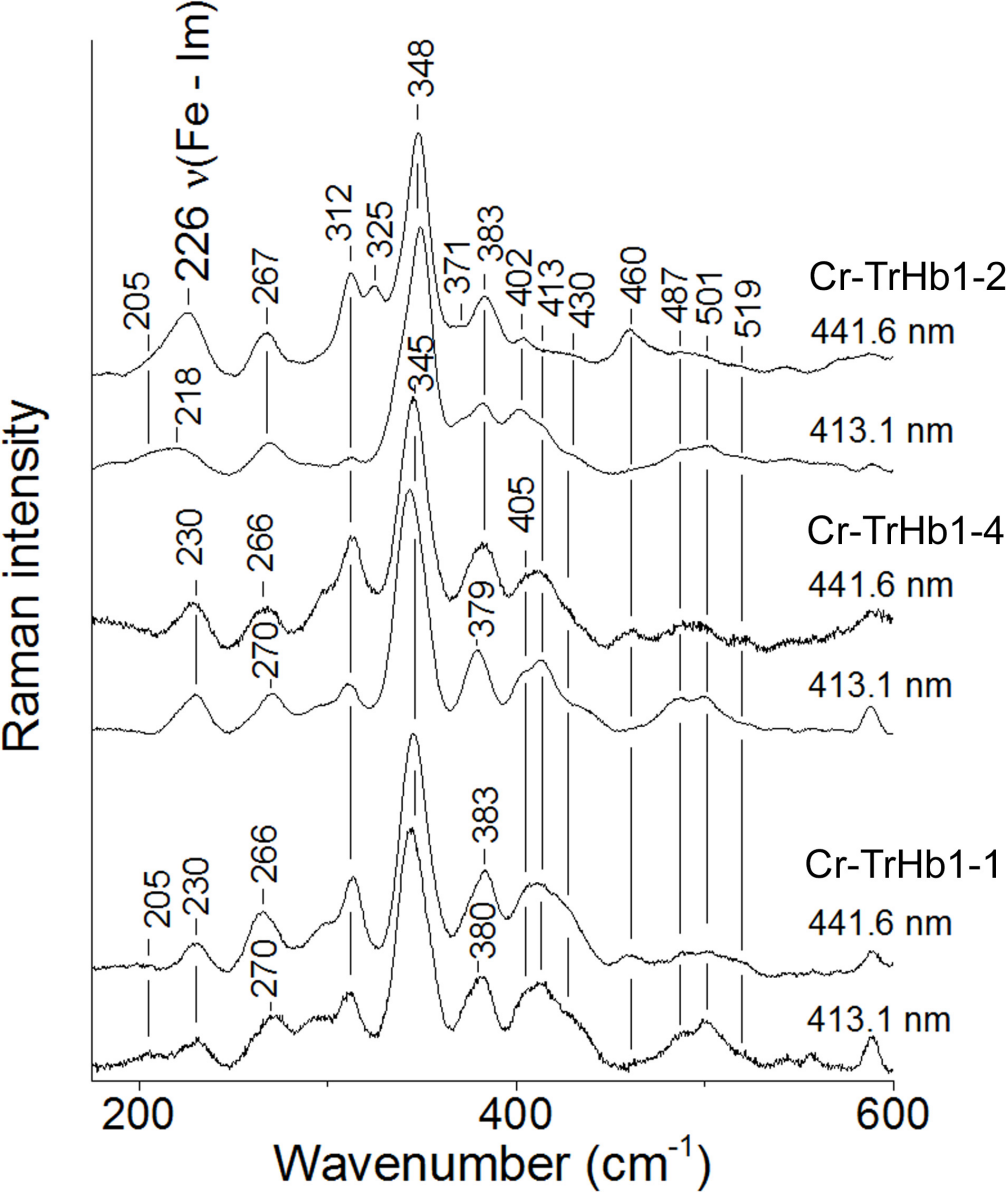
Resonance Raman spectra in the low-frequency region of ferrous Cr-TrHb1-1, Cr-TrHb1-2, and Cr-TrHb1-4. The resonance Raman spectra were obtained with the excitation wavelengths of 441.6 and 413.1 nm. Experimental conditions: 1 cm^−1^ resolution and 15 mW laser power at the sample; the total accumulation time was 30 min for Cr-TrHb1-2 and 20 min for Cr-TrHb1-1 and Cr-TrHb1-4. Spectra were obtained at pH 7.0 and 20°C. Spectra have been shifted along the ordinate axis to allow better visualization.

Absorption spectra of the unliganded reduced Fe(II) form of Cr-TrHb1-1, Cr-TrHb1-2, and Cr-TrHb1-4 are very similar to each other (Fig 4, Panel A) and they are characteristic of a 6cLS heme-Fe atom, as observed for the Fe(III) forms (see Fig 2), even though a small amount of a five-coordinate high-spin (5cHS) form occurs in Cr-TrHb1-2 (as indicated by the shoulders at 435 and 576 nm; see Fig 4, Panel A). This confirms the presence of a weaker sixth coordination heme-Fe ligand in Cr-TrHb1-2, as compared to Cr-TrHb1-1 and Cr-TrHb1-4, in agreement with the previous observations of the ferric form. Accordingly, in the high frequency RR spectra of Cr-TrHb1-2 two *v*_4_ modes are observed at 1352 cm^-1^ (corresponding to a 5cHS) and 1361 cm^-1^ (corresponding to a 6cLS) (Fig 4, panel B). Moreover, in the low frequency region, the *v*(Fe-Im) stretching mode at 226 cm^-1^ (Fig 5) is observed in the spectrum obtained with 441.6 nm excitation (*i*.*e*., in resonance with the Soret band of the 5cHS species at 435 nm), which disappears upon excitation with the 413.1 nm line. In fact, the *v*(Fe-Im) stretching mode is observed only in the 5cHS ferrous heme proteins [25] and it is enhanced in the RR spectra in resonance with the Soret band [26–28]. The frequency of the *v*(Fe-Im) stretching mode is significantly higher than that for human Hb (HbA) (214 cm^-1^) [25], but very close to that of many bacterial TrHbs [29–31], indicating a stronger heme-Fe-His bond.

**Fig 6:**
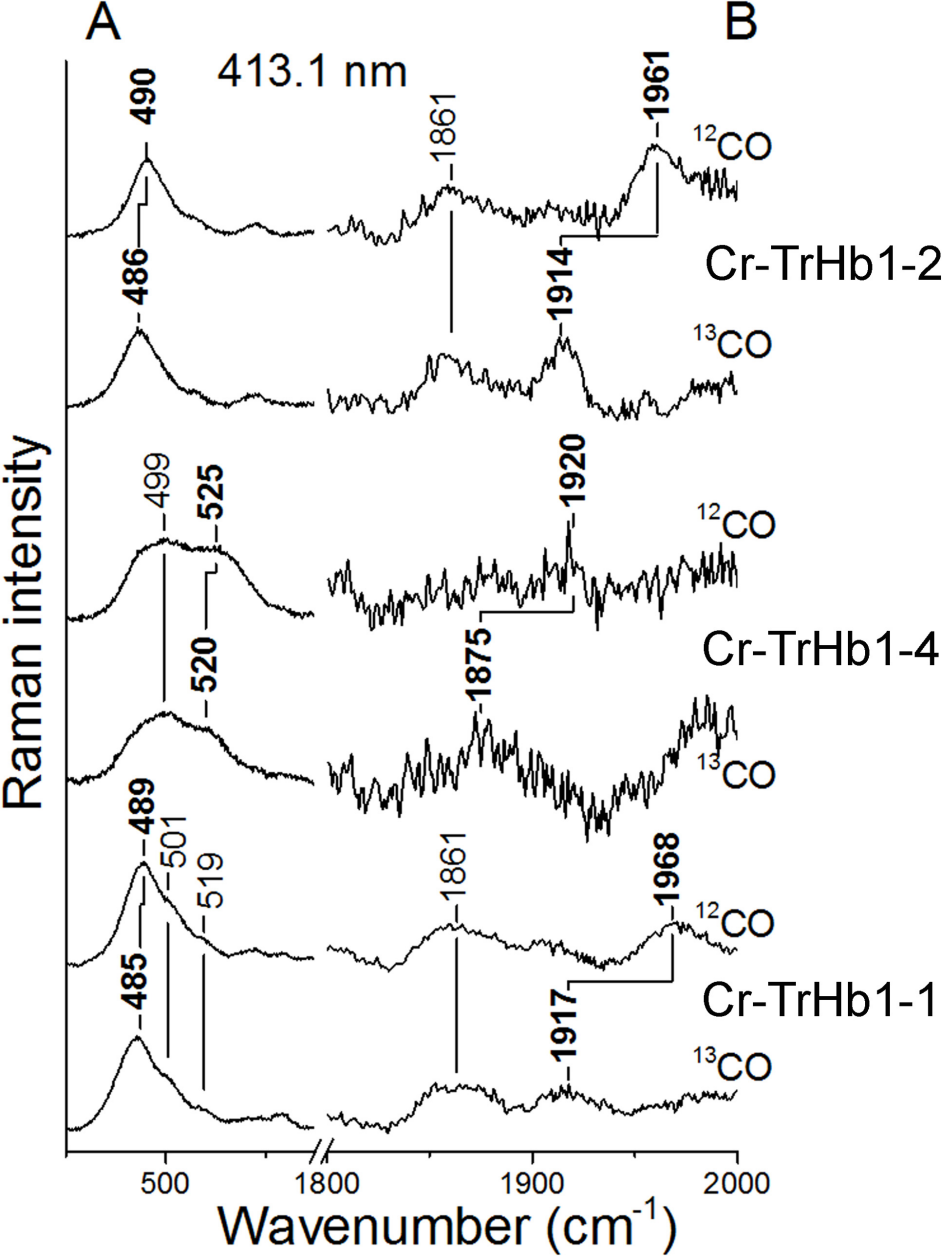
Resonance Raman spectra of the ^12^CO and ^13^CO complexes of ferrous Cr-TrHb1-1, Cr-TrHb1-2, and Cr-TrHb1-4. The resonance Raman spectra were obtained with excitation wavelength at 413.1 nm. *Panel A*, experimental conditions: 1 cm^−1^ resolution; Cr-TrHb1-1: 3 mW laser power at the sample and 20 min total accumulation time; Cr-TrHb1-2: 1.5 mW laser power at the sample and 10 min total accumulation time; and Cr-TrHb1-4: 0.3 mW laser power at the sample and 60 min total accumulation time. *Panel B*, experimental conditions: 3 cm^−1^ resolution; Cr-TrHb1-1: 3 mW laser power at the sample, and 60 min (^12^CO) and 20 min (^13^CO) total accumulation time; Cr-TrHb1-2: 1.5 mW laser power at the sample, and 40 min (^12^CO) and 100 min (^13^CO) total accumulation time; and Cr-TrHb1-4: 0.3 mW laser power at the sample, and 60 min (^12^CO) and 50 min (^13^CO) total accumulation time. The 1800–2000 cm^−1^ region has been expanded 10-fold with respect to the low frequency region. A cylindrical lens has been used to minimize the photolysis of the CO ligand induced by the laser light. Spectra have been shifted along the ordinate axis to allow better visualization. Spectra were obtained at pH 7.0 and 20°C.

Upon CO addition, ferrous Cr-TrHb1-1, Cr-TrHb1-2, and Cr-TrHb1-4 undergo carbonylation. In the RR spectra of the CO bound species (Fig 6) isotope-sensitive peaks were detected. Cr-TrHb1-1 and Cr-TrHb1-2 are characterized by a *v*(Fe-CO) stretching mode at 489 (for Cr-TrHb1-1) and 490 cm^-1^ (for Cr-TrHb1-2), which shift to 485 and 486 cm^-1^, respectively, in the presence of ^13^CO. Accordingly, the *v*(CO) stretching modes were identified at 1968 cm^-1^ (for Cr-TrHb1-1) and 1961 cm^-1^ (for Cr-TrHb1-2) (Fig 6, Panel B), which shifted to 1917 and 1914 cm^-1^, respectively, upon ^13^CO substitution. Conversely, Cr-TrHb1-4 is characterized by ν(Fe-CO) at 525 cm^-1^ (520 cm^-1^ in ^13^CO) and ν(CO) at 1920 cm^-1^ (1875 cm^-1^ in ^13^CO). Due to the very high photolysis even with very low laser power, the band at 1920 cm^-1^ is very weak.

**Fig 7:**
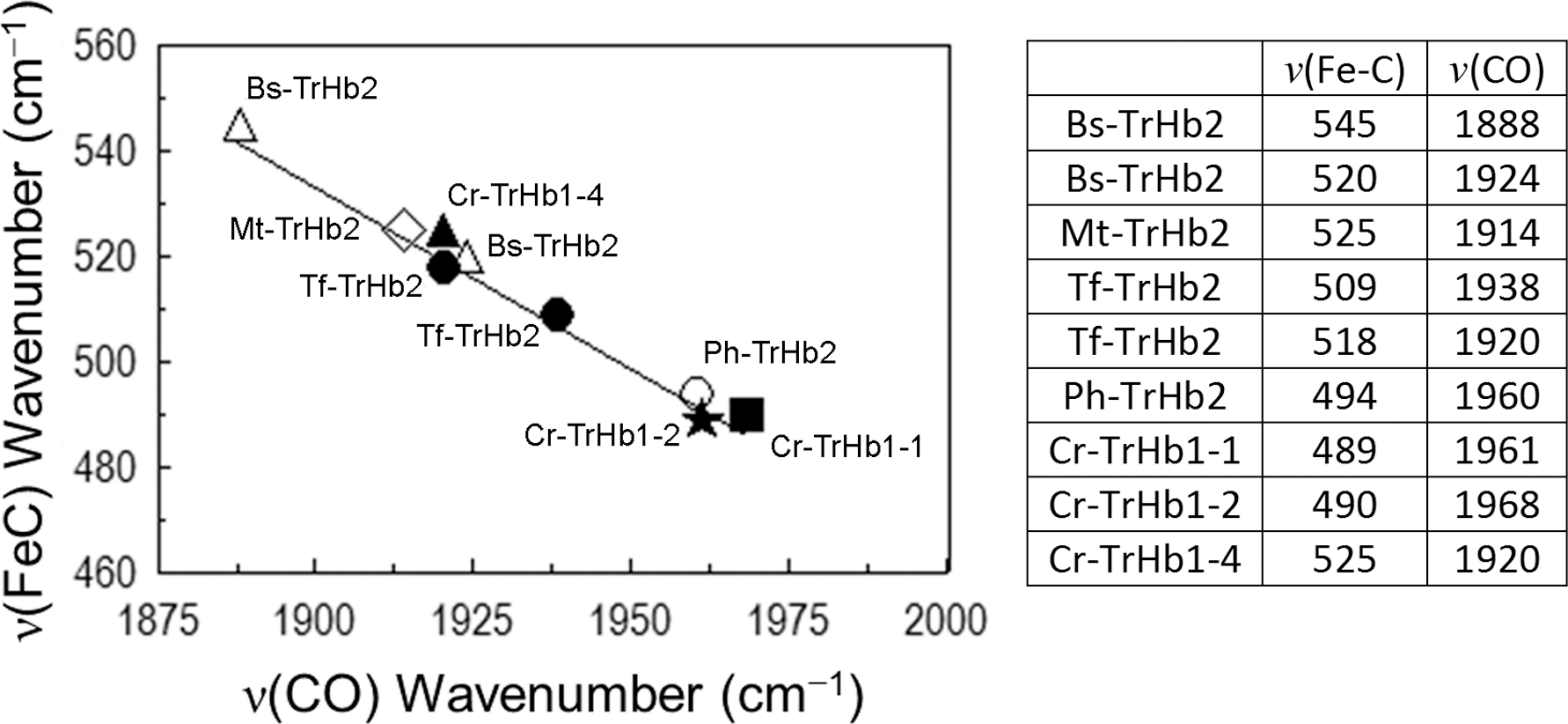
Correlation plot between the ν(Fe-C) and ν(C-O) frequencies for ferrous-CO complexes of Cr-TrHb1-1, Cr-TrHb1-2, and Cr-TrHb1-4, compared with class 2 TrHbs from *Bacillus subtilis* (Bs-TrHb2) [40], *Mycobacterium tuberculosis* (Mt-TrHb2) [41], *Pseudoalteromonas haloplanktis* (Ph-TrHb2) [31], and *Thermobifida fusca* (Tf-TrHb2) [30]. The frequencies are reported in the table. Data were obtained at pH 7.0 and 20°C.

CO is a sensitive probe for investigating distal environmental effects on ligand binding of heme-proteins [32]. In particular, polar interactions and the formation of H-bonds between the bound CO and the distal residues increase the extent of back-donation from the Fe d*π* to the CO π* orbitals. As a consequence, the Fe-C bond strengthens while the C-O bond weakens, thereby increasing the ν(Fe-C) vibrational frequencies and decreasing the ν(C-O) frequencies. A linear correlation with negative slope between the frequencies of the ν(Fe-C) and ν(C-O) stretching modes has been found for a large class of heme-protein-CO complexes, including bacterial TrHbs, and heme model compounds containing imidazole as the fifth heme-Fe ligand. The ν(Fe-C)/ν(C-O) position along the correlation line reflects the type and strength of distal polar interactions [32]. Fig 7 shows the plot of ν(Fe-C) *versus* ν(C-O) frequencies of different TrHbs, indicating that Cr-TrHb1-1 and Cr-TrHb1-2 are consistent with the presence of a linear Fe-CO unit with no or minimal polar interaction between the O atom and distal residues. On the other hand, Cr-TrHb1-4 frequencies fall in the region where the presence of a polar interaction with the oxygen atom of the bound CO occurs (Fig 7), suggesting the presence of a H-bond between the oxygen atom of CO and a distal residue.

### Ligand binding properties

#### Fe(III) form

**Fig 8:**
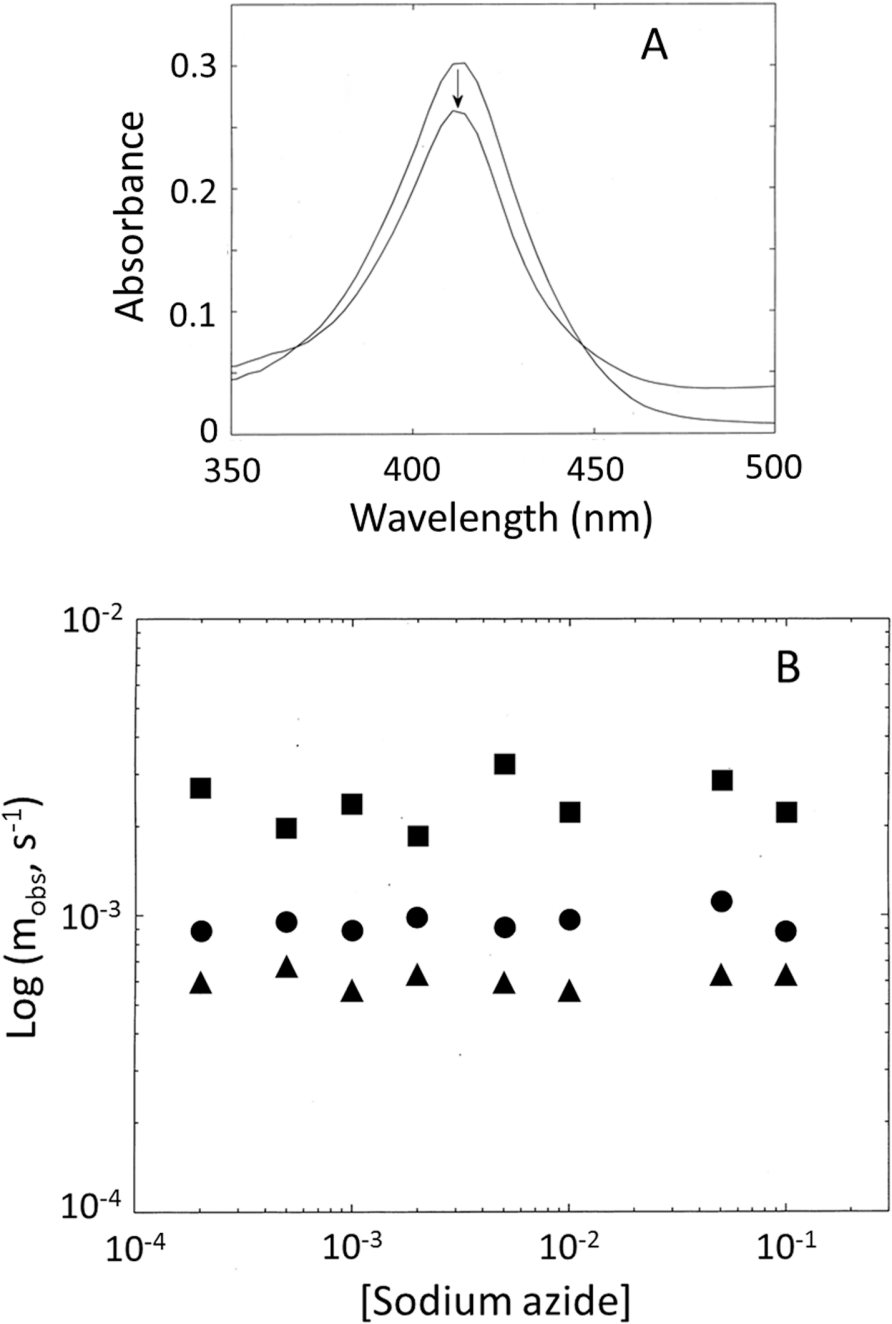
(*Panel A*) Absorption spectra of ligand-free and azide-bound ferric Cr-TrHb1-1. The sodium azide concentration was 0.1 M. The arrow indicates the azide-dependent spectroscopic transition. (*Panel B*) Azide concentration dependence of the observed rate *m*_*obs*_ for Cr-TrHb1-1 (●), Cr-TrHb1-2 (■) and Cr-TrHb1-4 (▲). Data were obtained at pH 7.0 and 20°C.

**Fig 9:**
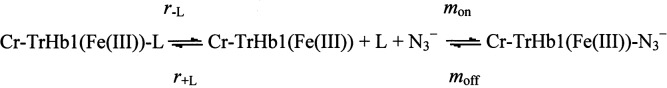
Scheme of the sodium azide binding reaction.

Fig 8 (panel A) shows, as an example, the spectral change following the reaction of Cr-TrHb1-1 with sodium azide at pH 7.0, which is characterized by a decrease of the extinction coefficient upon ligand binding without a significant change of the peak wavelength; the same behavior has been also observed for Cr-TrHb1-2 and Cr-TrHb1-4 (data not shown). This spectroscopic feature suggests that sodium azide binding indeed occurs, but there is no coordination change of the heme-Fe atom, the initial and final forms being both 6cLS, as also suggested by absorption and RR spectra (see Figs 2 and 3). The observation that binding kinetics are independent of sodium azide concentration confirms this interpretation (Fig 8, panel B), clearly indicating that the ligand-linked absorption change (Fig 8, panel A) is rate-limited by the dissociation of the sixth endogenous axial ligand (to which the concentration-independent rate constant must be referred), followed by a much faster sodium azide binding to Fe(III). This is illustrated in Fig 9 [33], where *r*_-L_ is the first-order dissociation rate of the endogenous sixth axial ligand L from the oxidized Fe(III) form, *r*_+L_ is the re-association rate for this endogenous ligand, which competes with the second-order association rate constant *m*_on_ of the exogenous ligand N_3_^-^, and *m*_off_ refers to the N_3_^−^ dissociation rate constant. The lack of any sodium azide concentration dependence means that, at each [N_3_^−^], *r*_*-L*_ << *m*_*on*_ [N_3_^-^].

The resulting rate constants for the dissociation of the endogenous sixth axial ligand from the ferric heme-Fe atom are 9.5(±1.2)×10^−4^ s^−1^ (for Cr-TrHb1-1), 2.5(±0.4)×10^−3^ s^−1^ (for Cr-TrHb1-2), and 5.6(±0.9)×10^−4^ s^−1^ (for Cr-TrHb1-4). The faster rate constant observed for Cr-TrHb1-2 agrees with the spectroscopic observation indicating the occurrence of a weaker endogenous ligand bound at the sixth coordination position of the heme-Fe(III) atom.

#### Fe(II) form

**Fig 10:**
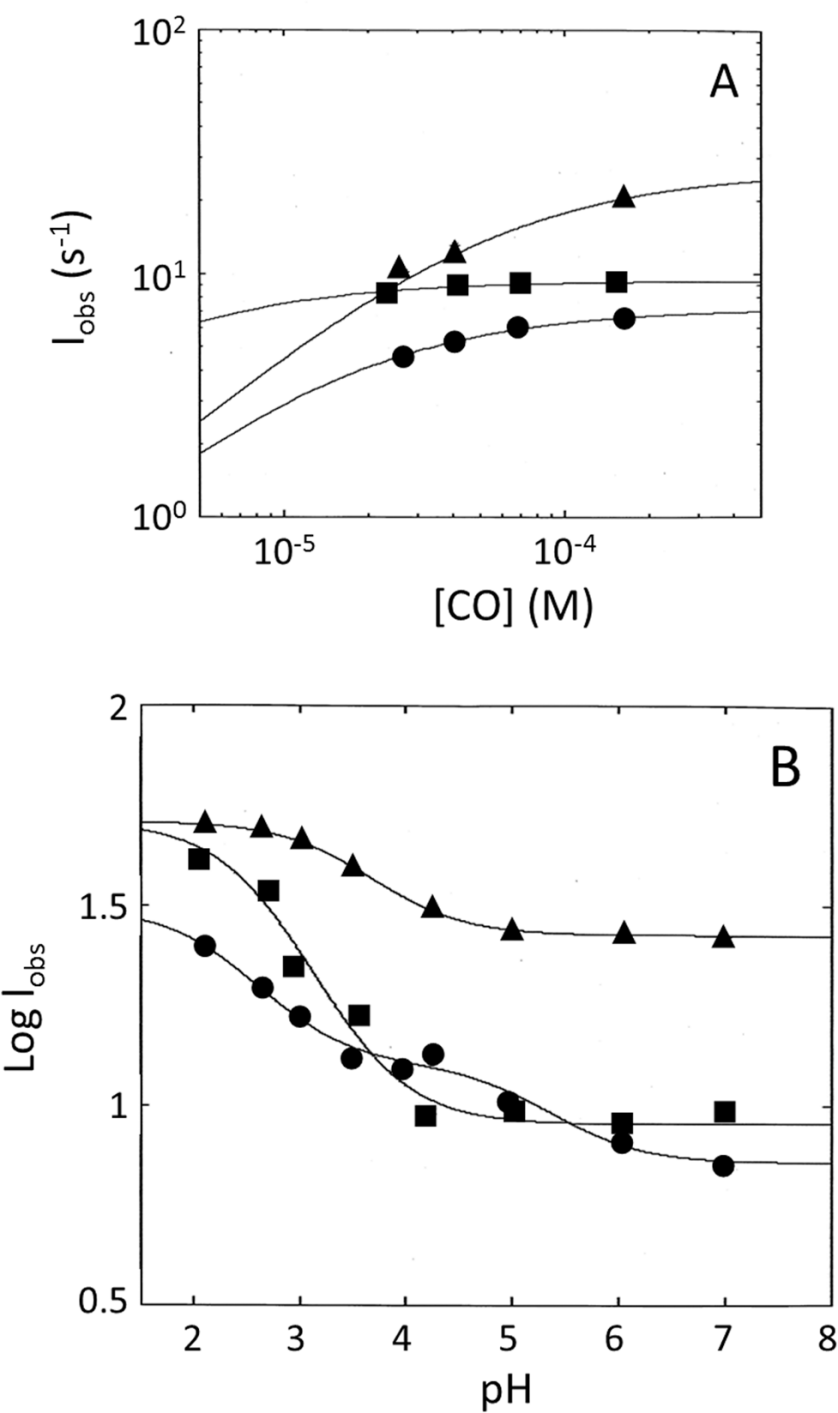
(*Panel* A) Effect of the CO concentration on the observed rate constant *l*_*obs*_ for Cr-TrHb1-1 (●), Cr-TrHb1-2 (■), and Cr-TrHb1-4 (▲) CO binding. Continuous lines have been obtained by non-linear least-squares fitting of data according to Eq (2C) with parameters reported in Table 1. Data were obtained at pH 7.0 and 20°C. (*Panel B*) pH dependence of the observed rate constant *l*_*obs*_ for Cr-TrHb1-1 (●), Cr-TrHb1-2 (■) and Cr-TrHb1-4 (▲) CO binding. Continuous lines have been obtained by non-linear least-squares fitting of data according to Eq (3) with parameters reported in Table 1. Data were obtained at 20°C.

**Fig 11:**
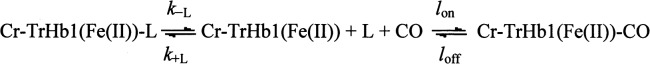
Scheme of the CO binding reaction.

Cr-TrHb1-1, Cr-TrHb1-2, and Cr-TrHb1-4 display CO binding kinetics characterized by a ligand concentration dependence (Fig 10, panel A), all of which however tend to level off at [CO] ≥ 50 μM, indicating the existence of a rate-limiting step at high CO concentrations (Fig 10, panel A). Such a behavior can be accounted for by Fig 11 [33], where *k*_−L_ is the first-order dissociation rate of the endogenous sixth axial ligand L from the reduced Fe(II) form, *k*_+L_ is the re-association rate for this endogenous ligand, which competes with the second-order association rate constant *l*_on_ of the exogenous ligand CO, and *l*_off_ refers to the CO dissociation rate constant. Therefore, the process can be described by Eqs 2A–2C:

$$l_{obs}=\frac{k_{-L}\cdot l_{on}\cdot \left[CO\right]}{k_{+L}\cdot \left[L\right]+l_{on}\cdot \left[CO\right]}+\frac{l_{off}\cdot k_{+L}\cdot \left[L\right]}{k_{+L}\cdot \left[L\right]+l_{on}\cdot \left[CO\right]}$$ (Equation 2A)

which becomes, since [*L*] is constant (being an endogenous ligand of the protein), ^c^ *k*_+L_ = *k*_+L_ · [L] and

lobs=k−L⋅lon⋅[CO]kc+L+lon⋅[CO]+loff⋅kc+Lkc+L+lon⋅[CO] (Equation 2B)

Eq (2B) can be simplified to

lobs=k⋅−Lτ⋅[CO]1+τ⋅[CO]+loff1+τ⋅[CO] (Equation 2C)

where *τ* (= *l*_on_ / ^c^ *k*_+L_) represents the competition between the association of the endogenous ligand L and the exogenous CO. Hence, the leveling off of the CO binding kinetics (see Fig 10, panel A) corresponds to the rate-limiting constant *k*_-L_, which occurs when *τ* [CO] >> 1 (see Eq 1C), since in the second term of Eq 1C the value of *l*_off_ is usually very small (as in our case, being *l*_off_ = 9(±2)×10^−3^ s^−1^ for all Cr-TrHb1s; data not shown). All parameters used for the analysis of the data shown in Fig 10A are reported in Table 1. Values of the rate-limiting steps differ slightly for the three Cr-TrHb1s, being 7.2(±1.1) s^−1^ for Cr-TrHb1-1, 9.3(±1.9) s^−1^ for Cr-TrHb1-2, and 2.7(±3.6)×10^1^ s^−1^ for Cr-TrHb1-4. The different values of the rates limiting CO binding likely reflect different sixth ligand bond strengths of the heme-Fe(II) atom of the Cr-TrHb1s. Since structural modeling (Fig 1) seems to indicate that in all three Cr-TrHb1s a Lys residue might be the sixth ligand, as suggested for Cr-TrHb1-1 by others [16], this difference can only be attributed to a somewhat different structural arrangement of the heme pocket in the three Cr-TrHb1s, which is also evident from the structural modeling (see Fig 1).

**Table 1:** Kinetic parameters for CO binding to and O_*2*_ dissociation from Cr-TrHb1s.

	Cr-TrHb1-1	Cr-TrHb1-2	Cr-TrHb1-4
**CO binding at pH 7.0 and 20°C**			
*k*_-L_ (s^-1^)	7.2±1.1	9.3±1.9	2.6(±0.4)×10^1^
*l*_off_ (s^-1^)	1.1(±0.2)×10^−2^	8.7(±1.1)×10^−3^	9.7(±1.9)×10^−3^
*τ*	6.6(±0.8)×10^4^	4.1(±0.7)×10^5^	2.0(±0.4)×10^4^
**Parameters fitting the pH-dependence of CO binding at 20 C** ^a^			
*l*_*0*_ (s^-1^)	7.2±1.1	9.3±1.9	2.6(±0.4)×10^1^
*l*_*1*_ (s^-1^)	1.3(±0.3)×10^1^	5.1(±0.6)×10^1^	5.1(±0.7)×10^1^
*l*_*2*_ (s^-1^)	3.1(±0.5)×10^1^	-	-
*pK*_*a1*_	5.3±0.2	2.8±0.2	3.5±0.2
*pK*_*a2*_	2.4±0.2	-	-
**Oxygen Pulse at pH 7.0 and 20°C**			
*k*_*1*_ (s^-1^)	1.1(±0.2)×10^1^	8.2(±0.1)×10^0^	1.7(±0.3)×10^1^
*k*_*2*_ (s^-1^)	1.6(±0.3)×10^−1^	1.5(±0.3)×10^–1^	4.1(±0.7)×10^−2^

^a^Values obtained at [CO] = 1.5×10^–4^ M after mixing.

As shown in Fig 10B, values of the CO binding rate constants for Cr-TrHb1-1, Cr-TrHb1-2, and Cr-TrHb1-4 increase upon lowering pH from 7.0 to 2.0. This behavior has been studied at a constant CO concentration (*i*.*e*., 150 μM), corresponding to a situation where the rate-limiting step is operative at pH 7.0, even though we cannot rule out the possibility that lowering the pH brings about the cleavage of the axial ligand(s) bond(s), rendering the reaction bimolecular. The pH-dependence of the pseudo-first-order rate constant *l*_*obs*_ has been analyzed according to Eq 3:

$$l_{obs}=\sum_{i=0}^{i=n}l_{i}\cdot \frac{\prod_{r=0}^{r=i}K_{ar}\cdot {\left[H^{+}\right]}^{i}}{\sum_{i=0}^{i=n}K_{ai}\cdot {\left[H^{+}\right]}^{i}}$$ (Equation 3)

where *n* is the number of protonating groups, *i* is the number of protonated groups, *K*_*ai*_ is the proton binding affinity of the *i*th protonated group (*i*.*e*., *K*_*ai*_ = 10^*pKai*^), and [*H* ^*+*^] (= 10^*-pH*^) is the proton concentration.

The pH-dependence of the CO binding rate constant for Cr-TrHb1-1 is characterized by two protonation events (displaying p*K*_*a1*_ = 5.3±0.2 and p*K*_*a2*_ = 2.4±0.2) (see Fig 10, panel B), possibly reflecting the protonation of the distal and the proximal axial ligands, respectively. This being the case, it would mean that at pH < 4.5 the heme is penta-coordinated and CO binding occurs according to a bimolecular reaction. On the other hand, the pH-dependence of the CO binding rate constant for both Cr-TrHb1-2 and Cr-TrHb1-4 seems to be characterized by only one protonation event (with p*K*_*a*_ = 2.8±0.2 for Cr-TrHb1-2 and p*K*_*a*_ = 3.5±0.2 for Cr-TrHb1-4; see Fig 10, panel B), likely referable to the protonation of the proximal histidine [34,35]. An additional feature, which distinguishes Cr-TrHb1-1 from Cr-TrHb1-2 and Cr-TrHb1-4, is the asymptotic value of the rate at very low pH, which is closely similar for Cr-TrHb1-2 and Cr-TrHb1-4 (= 5.1(±6.2)×10^1^ s^-1^; see Fig 10, panel B), whereas it is significantly slower for Cr-TrHb1-1 (= 3.1(±4.5)×10^1^ s^-1^); it likely reflects a different energetic barrier for the CO ligand access into the heme pocket of the different Cr-TrHb1s. This is consistent with he possibility of distinct ligand access pathways to the heme pocket identified in the structural models of the three Cr-TrHb1s (see S1 Fig).

### Oxygen pulse

**Fig 12:**
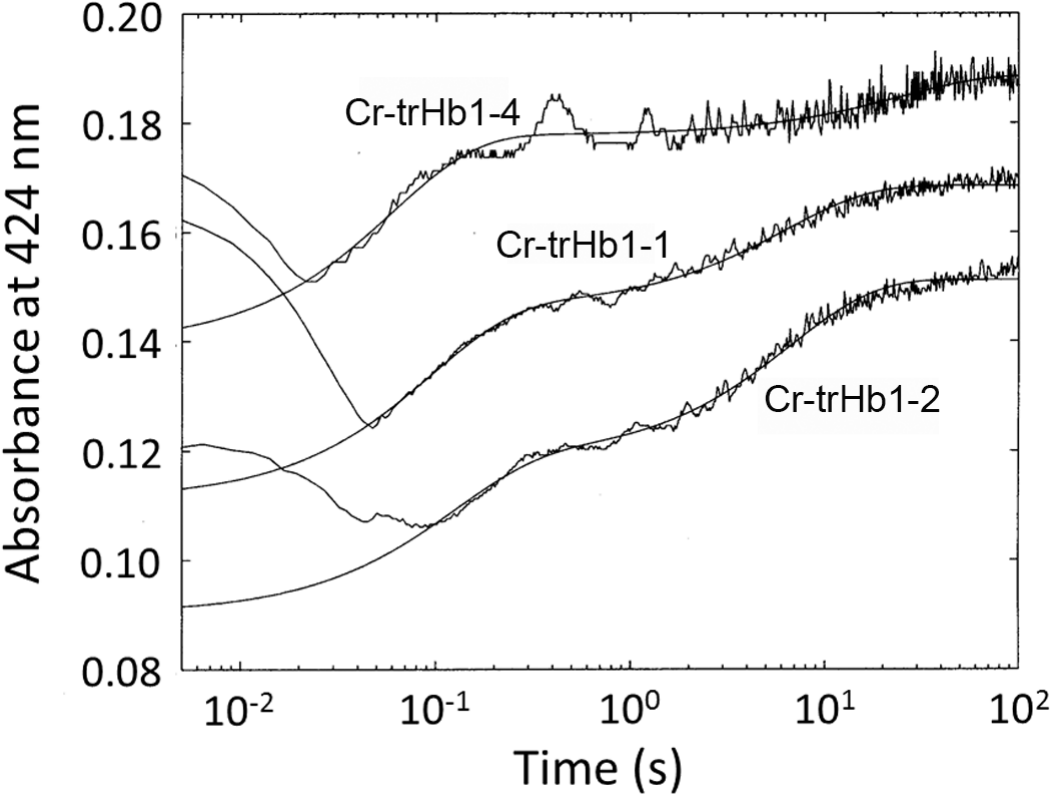
Time course of the absorption spectral changes at 424 nm after an oxygen pulse for Cr-TrHb1-1, Cr-TrHb1-2 and Cr-TrHb1-4 (as indicated). Continuous lines correspond to the non-linear least-squares fitting of data according to Eq (1), employing parameters reported in Table 1. Data were obtained at pH 7.0 and 20°C.

Cr-TrHb1-1, Cr-TrHb1-2, and Cr-TrHb1-4 bind reversibly O_2_, as indicated by the O_2_ pulse experiment, where the initial absorption decrease at 424 nm indeed reflects the very fast O_2_ binding (Fig 12); the subsequent absorption increase at 424 nm reflects the release of O_2_ from the heme-Fe(II)-O_2_ at a rate corresponding to the O_2_ dissociation rate constant (see Fig 12). It is evident that all three Cr-TrHb1s display a biphasic O_2_ dissociation process with markedly different rate constants (Fig 12 and Table 1). Such a behavior can be referred to the presence of two populations in the transient ferrous oxygenated forms, which either are not in equilibrium or else display equilibration rate constants much slower than those of the O_2_ dissociation rate constants. This feature might also reflect a different distal structural arrangement between the two populations, which are characterized by significantly different energy barriers for the ligand exit pathway. This is also suggested by structural modeling study (see S1 Fig), which shows that the three Cr-TrHb1s may be characterized by different tunnels for the ligand migration toward and/or from the heme pocket.

### Nitrite reductase activity

In order to study the possibility that Cr-TrHb1-1, Cr-TrHb1-2, and Cr-TrHb1-4 display an enzymatic activity related to NO metabolism, their nitrite reductase activity has been investigated between pH 5.0 and pH 9.0.

**Fig 13:**
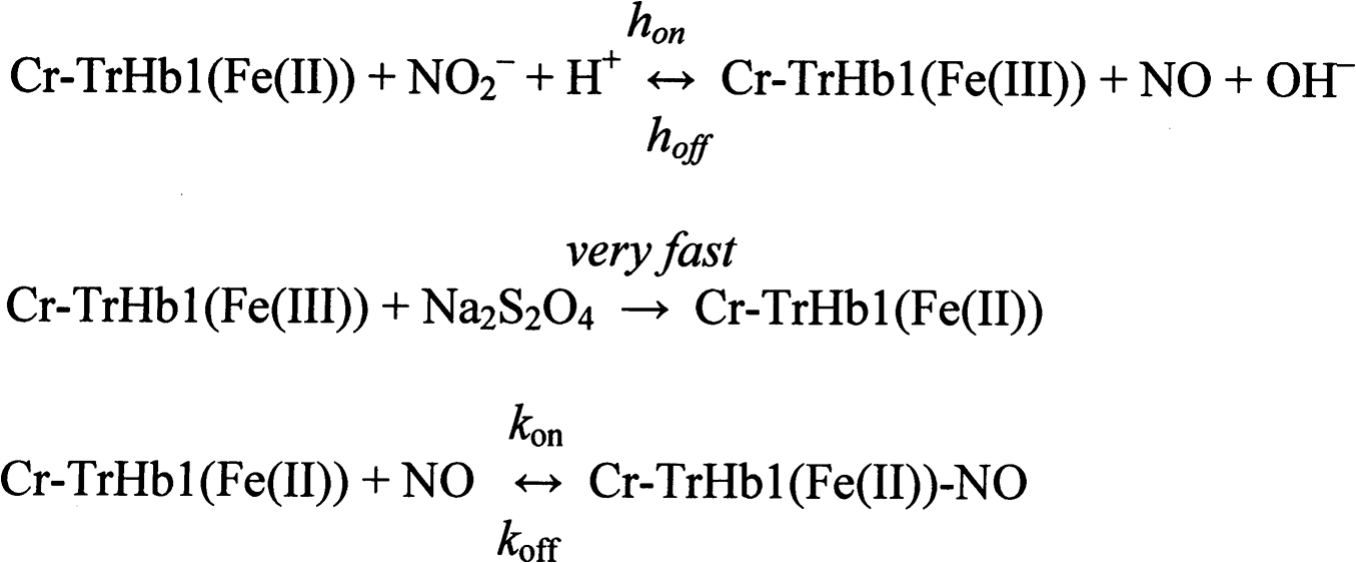
Scheme of the Nitrite-reductase reaction.

In general terms, the nitrite reductase activity catalyzed by heme-proteins [36,37] can be described according to Fig 13.

In this reaction the rate-limiting step is represented by the rate of the first step *h*_*on*_, thus allowing to establish the bimolecular rate constant for the interaction of Cr-TrHb1-Fe(III) with NO_2_^−^, according to Eq 4:

$$h_{obs}=h_{on}\cdot \left[NO_{2}^{-}\right]+h_{off}$$ (Equation 4)

with *h*_*off*_ ≈ 0, since the first step of the reaction is essentially irreversible.

**Fig 14:**
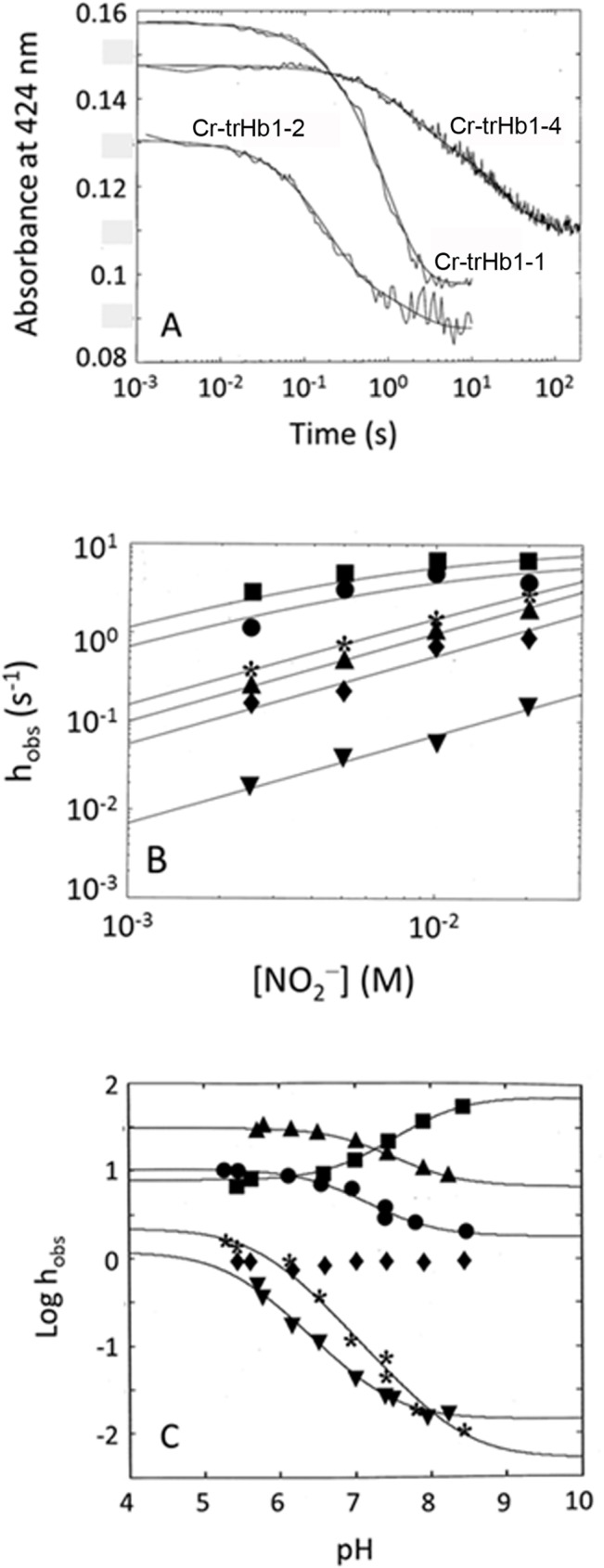
(*Panel A*) Time course of the absorption spectral changes at 424 nm for the reaction of Cr-TrHb1-1 (●), Cr-TrHb1-2 (■) and Cr-TrHb1-4 (▲) with NO_*2*_^−^. The sodium nitrite concentration was 10 mM. Continuous lines correspond to the non-linear least-squares fitting of data according to Eq 1, employing parameters reported in Table 2. (*Panel B*) NO_*2*_^−^ concentration dependence of the observed rate constants *h*_*obs*_ for Cr-TrHb1-1 (● for the fast phase, * for the slow phase), Cr-TrHb1-2 (■ for the fast phase, ♦ for the slow phase), and Cr-TrHb1-4 (▲ for the fast phase, ▼ for the slow phase) catalyzed conversion of NO_*2*_^−^ to NO. The continuous lines have been obtained by non-linear least-squares fitting of data according to Eqs (2C) and (4) with parameters reported in Table 2. Data were obtained at pH 6.6 and 20°C. (*Panel C*) pH dependence of the observed rate constant *h*_*obs*_ for the conversion of NO_*2*_^−^ to NO by Cr-TrHb1-1 (● for the fast phase, * for the slow phase), Cr-TrHb1-2 (■ for the fast phase, ♦ for the slow phase), and Cr-TrHb1-4 (▲ for the fast phase, ▼ for the slow phase). The continuous lines have been obtained by non-linear least-squares fitting of data according to Eq 3 with parameters reported in Table 2. Data were obtained at 20°C.

However, unlike other heme proteins (such as horse heart myoglobin and human serum heme-albumin) [36,37], all three Cr-TrHb1s display a biphasic nitrite reductase process (Fig 14, panels A and B) at all investigated pH values (Fig 14, panel C). It is noteworthy that, at pH 6.6, the NO_2_^−^ concentration dependence of the two phases differs; in particular, the faster process displays a concentration dependence characterized by the leveling off at high NO_2_^−^ concentrations, as observed for CO (Fig 10, panel A and Fig 14, panel B), whereas the slower process follows an apparently bimolecular behavior (Fig 14, panel B). Clearly, it does not rule out the possibility of a leveling off also of this phase at [NO_2_^−^] much higher than those investigated. Therefore, the slow phase has been analyzed according to Eq 4, whereas the fast phase, being characterized by a rate-limiting step (as in the case of CO, see Fig 9) has been analyzed according to Eq 5:

hobs=k⋅−Lρ⋅[NO2−]1+ρ⋅[NO2−]+hoff1+ρ⋅[NO2−] (Equation 5)

Eq 5 is conceptually equal to Eq 2C, where *k*_-L_ has the same meaning (and the same value) as above and *ρ* (= *h*_on_ / ^c^ *k*_+L_) represents the competition between the association of the endogenous axial ligand and the exogenous ligand NO_2_^−^; the second term in Eq 5 has a value very close to 0, since, as stated above, *h*_off_ ≈ 0 [36,37].

Analysis of the parameters reported in Table 2 allows the following considerations to be made: (*i*) while for Cr-TrHb1-1 and Cr-TrHb1-4 both phases are markedly pH-dependent, in the case of Cr-TrHb1-2 only the fast phase appears to be pH-dependent, the slowest one being essentially pH-independent (Fig 14, panel C); (*ii*) *pK*_*af*_ values range around 6.7–7.0 and they are always higher than *pK*_*as*_, which are in the range of 5.4–5.7 (see Table 2); (*iii*) in the case of Cr-TrHb1-2, which displays only *pK*_*af*_ = 8.0±0.2, this value is significantly higher than that for Cr-TrHb1-1 and Cr-TrHb1-4, clearly suggesting a different protonating group involved in the pH-dependence; (*iv*) on the basis of literature data [36.37], the rates of both the slow and fast reductive nitrosylation phases of Cr-TrHb1-1 and Cr-TrHb1-4 increase upon decreasing pH around neutrality, meaning that this (pseudo-)enzymatic reaction requires one proton for NO and OH^–^ formation (see Fig 11); (*v*) the rate of the fast reductive nitrosylation phase of Cr-TrHb1-2 decreases upon decreasing pH, and the rate of the slow phase of Cr-TrHb1-2 reductive nitrosylation is pH-independent, a result never observed for the reductive nitrosylation of other heme-proteins [36,37]. In this respect, it is worth remarking that the similar pH-dependence of human cytoglobin reductive nitrosylation has been interpreted accounting for the reversible pH-dependent penta-to-hexa-coordination transition of the heme-Fe(II) atom [38]. This interpretation could be applied also to Cr-TrHb1-2, since the RR spectra indeed suggest that a portion of Cr-TrHb1-2 might be penta-coordinated in the unliganded Fe(II) form (Figs 4 and 5); (*vi*) values of *h*_on_ for the nitrite reductase activity of Cr-TrHb1-1, Cr-TrHb1-2, and Cr-TrHb1-4 (see Table 2) are similar to those reported for *Synecocystis* Hb, *Arabidopsis thaliana* Hb class 1, and rice non-symbiotic Hb class 1, which are more than 5 times faster than those observed for animal Hbs [12,39].

**Table 2:** Kinetic parameters for the nitrite reductase activity of the Cr-TrHb1s.

	Cr-TrHb1-1	Cr-TrHb1-2	Cr-TrHb1-4
**Parameters of the nitrite reductase activity pH 6.6 and 20°C**			
***Fast Process***			
*k*_-L_ (s^-1^)	7.2±1.1	9.3±1.9	2.6(±0.4)×10^1^
*ρ*	1.0(±0.3)×10^2^	1.4(±0.4)×10^2^	5.7±0.9
***Slow Process***			
*h*_on_ (M^-1^ s^-1^)	9.8(±3.4)×10^1^	5.4(±1.7)×10^1^	6.9±2.4
**Parameters fitting the pH-dependence of nitrite reductase activity at 20°C** ^a^			
^*f*^ *h*_*0*_ (s^-1^)	1.8±0.3	7.0(±1.2)×10^1^	6.6±1.0
^*f*^ *h*_*1*_ (s^-1^)	1.0(±0.2)×10^1^	7.8(±1.3)	3.1(±0.7)×10^1^
^*s*^ *h*_*0*_ (s^-1^)	5.1(±0.8)×10^−3^	8.6(±2.1)×10^−1^	1.4(±0.3)×10^−2^
^*s*^ *h*_*1*_ (s^-1^)	2.2±0.4	-	1.2±0.3
*pK*_*af*_	6.8±0.2	8.0±0.2	7.2±0.2
*pK*_*as*_	5.8±0.2	-	5.4±0.2

^a^ Values obtained at [NO_2_^-^] = 5.0×10^–2^ M after mixing.

On the whole, these observations suggest that Cr-TrHb1-1, Cr-TrHb1-2, and Cr-TrHb1-4 could take part in the finely regulated processes for the assimilation of inorganic nitrogen, representing a possible nitrite-dependent NO source. Reactive nitrogen species (RNS) are present in and around photosynthetic algae and cyanobacteria as products of enzymes involved in nitrite or nitrate reduction. Thus, a further conceivable function of Cr-TrHb1-1, Cr-TrHb1-2, and Cr-TrHb1-4 in *C*. *reinhardtii* may be related to the regulation of deoxygenation and of RNS level.

## Concluding Remarks

The three TrHbs from *C*. *reinhardtii* (*i*.*e*., Cr-TrHb1-1, Cr-TrHb1-2, and Cr-TrHb1-4) display a 6cLS form both in the Fe(III) and in the Fe(II) species. However, the strength of the axial hexa-coordinating bond is redox-linked, since it is much stronger in the Fe(III) species, rendering the rate constant of sodium azide binding independent of the ligand concentration. On the other hand, the axial hexa-coordination of the Fe(II) form is much weaker, as indicated by the exogenous ligands (*e*.*g*., CO and NO_2_^−^) binding, even though it is rate-limited at high ligand concentrations. It is interesting to point out that this strength may be correlated with the speed of the rate-limiting step (likely corresponding to the dissociation of the hexa-coordinating endogenous ligand), which is different for the three Cr-TrHb1s, displaying at pH 6.6–7.0 the fastest value for Cr-TrHb1-4 (= 2.4(±0.4)×10^1^ s^−1^) and the slowest value for Cr-TrHb1-1 (= 7.2±1.1 s^−1^). This three-fold difference of the rate-limiting step (whose value is the same for a given TrHb1, independent of the ligand, as shown in Tables 1 and 2) corresponds to a difference of about 3 kJ/mole for the bond strength, likely reflecting the possibility of a different tension on the axial bond due to conformational differences among the three Cr-TrHb1s.

Upon lowering the pH, CO binding and nitrite reductase activity show drastically different proton-linked groups involved in the modulation of the reaction, clearly indicating that the two processes are regulated by different residues. Thus, for CO binding the reactivity is mostly modulated by groups with very low *pK*_*a*_ values (ranging between 2.4 and 3.5, see Table 1), likely reflecting the protonation of the proximal histidine, and only Cr-TrHb1-1 displays an additional functionally relevant residues with a *pK*_*s*_ = 5.3±0.2 (see Table 1). On the other hand, for the fast-reacting species catalyzing the conversion of NO_2_^−^ to NO, *pK*_*a*_ values are much higher (ranging between 6.7 and 8.0, see Table 2) while they are much lower for the slow-reacting species (ranging between 5.4 and 5.8, see Table 2), being fairly close to that of Cr-TrHb1-1 for CO binding (see Table 1). In this respect, it should be noted that, in spite of the different pK_a_ values (see Tables 1 and 2 for CO and NO_2_^−^, respectively), the higher asymptotic values for the observed rates are fairly similar, being faster for Cr-TrHb1-2 and Cr-TrHb1-4 and slower for Cr-TrHb1-1. Indeed, the difference of pK_a_ values for the two ligands of the Fe(II) form might simply reflect the fact that the mode of binding and the reaction mechanism are different, involving different residues in the modulation of the proton-linked behavior.

The functional behavior here described could have a considerable metabolic significance in view of the recently assessed importance of the NO/NO_2_^−^ equilibrium for nitrate and ammonium assimilation in *C*. *reinhardtii* [18], even though this role should be assessed from *in vivo* experiments on the hypoxic survival, as tested for Cr-TrHb1-8 [16]. Thus, variations of the environmental pH appear to affect to a large extent the nitrite reductase activity of these Cr-TrHb1-1, Cr-TrHb1-2, and Cr-TrHb1-4, to which the NO/NO_2_^−^ metabolism is closely related, consequently altering the uptake of ammonium. In fact, the three Cr-TrHb1-1, Cr-TrHb1-2, and Cr-TrHb1-4 display some slight differences in the redox-linked reactivity and spectroscopic properties, possibly reflecting a different nature and geometry of the heme distal residues. This raises the question of whether the simultaneous presence of globins in the same organism might be related to various regulatory roles in different metabolic routes, acquiring functional relevance also in view of the assessed metabolic role for Cr-TrHb1-8 [16].

## Supporting Information

S1 Fig Schematic representation of the molecular models of Cr-TrHb1-1, Cr-TrHb1-2, and Cr-TrHb1-4. Note the *N*-terminal extensions of Cr-TrHb1-2 and Cr-TrHb1-4 which partially hinder access to the heme distal cavity.
(TIF)
