# Supplementary figures and images for: Functional and Spectroscopic Characterization of Chlamydomonas reinhardtii Truncated Hemoglobins
Source: PLoS One. 2015 May 20;10(5):e0125005. doi: 10.1371/journal.pone.0125005 (PMC4439042; doi:10.1371/journal.pone.0125005)

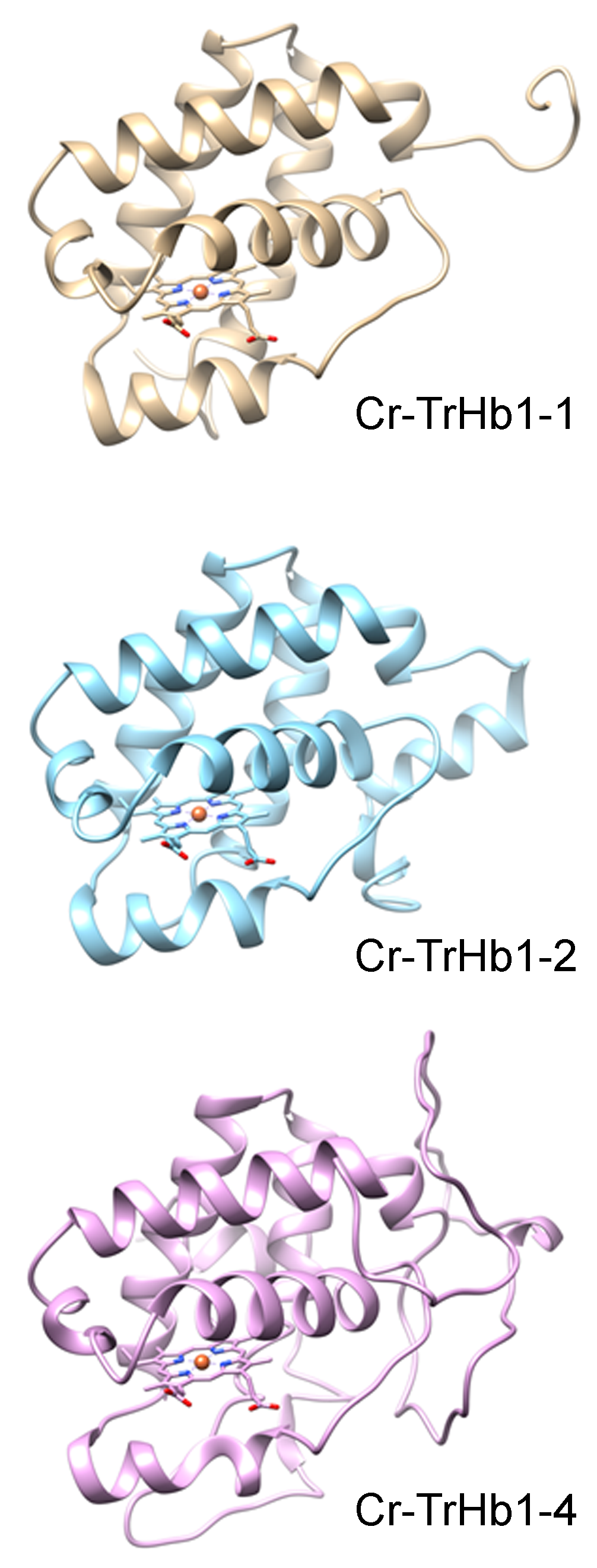

Supplement: S1 Fig — Note the N-terminal extensions of Cr-TrHb1-2 and Cr-TrHb1-4 which partially hinder access to the heme distal cavity. (TIF) [file pone.0125005.s001.tif]
